# On the Current Drive Capability of Low Dimensional Semiconductors: 1D versus 2D

**DOI:** 10.1186/s11671-015-1134-6

**Published:** 2015-10-29

**Authors:** Y. Zhu, J. Appenzeller

**Affiliations:** Birck Nanotechnology Center, Purdue University, West Lafayette, IN 47907 USA

**Keywords:** Low-dimension semiconductor, Electron transport, Current drive capability

## Abstract

Low-dimensional electronic systems are at the heart of many scaling approaches currently pursuit for electronic applications. Here, we present a comparative study between an array of one-dimensional (1D) channels and its two-dimensional (2D) counterpart in terms of current drive capability. Our findings from analytical expressions derived in this article reveal that under certain conditions an array of 1D channels can outperform a 2D field-effect transistor because of the added degree of freedom to adjust the threshold voltage in an array of 1D devices.

**Electronic supplementary material:**

The online version of this article (doi:10.1186/s11671-015-1134-6) contains supplementary material, which is available to authorized users.

## Background

The trend of scaling CMOS technology toward ever smaller dimensions has resulted in device structures that resemble nanowires in terms of their cross-sectional dimensions, i.e., FinFETs and TriGates [1–6] are approaching heights and widths of few tens of nanometers. Depending on the nature of the channel material, and in particular if materials other than silicon are considered, size quantization effects can be relevant [7, 8] in these types of structures. Envisioning that the current trend of miniaturization prevails, one-dimensional modes will ultimately carry the current from source to drain. In other words, in order to continue channel length scaling, low-dimensional channel structures are introduced at the expense of lower current drive capabilities per wire. To compensate for the loss of material that is introduced by separating the individual wire structures, arrays of the same have to be built. The obvious question arising in this context is “Under which circumstances does this approach make sense and when does it fail or – as we will show below – under which conditions is it desirable to operate in the one-dimensional transport mode regime even without requiring the additional benefit of channel length scaling” [9–13].

To shine some light on these questions, we have studied a model system that consists of a two-dimensional gated channel with ideal source/drain contacts operating in the ballistic regime. This system is then “patterned” into individual one-dimensional channels of various dimensions and spacing between them. Note that the structures under consideration remain planar and do not provide the added advantage of effectively increasing the device width in the vertical direction (as in the case of FinFETs and TriGates). A comparison between both the on- and off-state performance of the various systems when operating in the quantum capacitance limit, i.e., the conduction and valence bands of the structure are under ideal gate control, reveals the desired operation window for low-dimensional nanowire arrays which goes beyond the arguments that typically motivate the introduction of FinFETs and TriGates.

## Methods

**Fig. 1:**
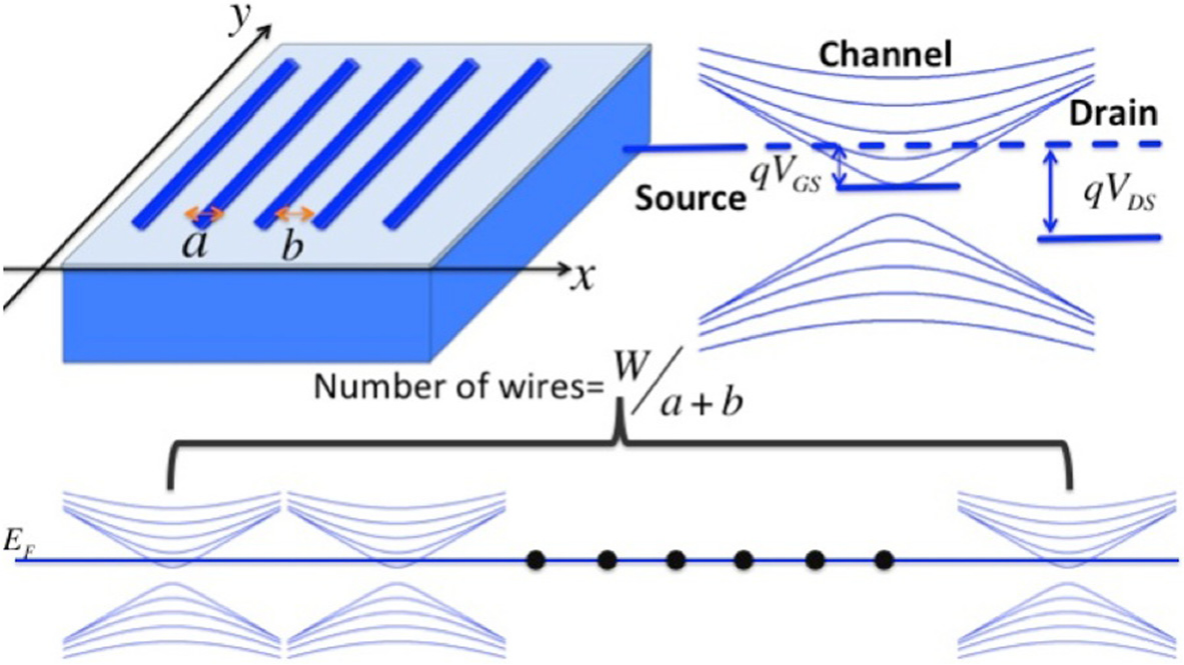
Model system (*top left*), impact of V_DS_ and V_GS_ on the 1D mode system (*top right*), and visualization of parallel conduction in an array of 1D wires (*bottom*)

Let us consider an array of 1D nanowires with width *a* that is separated by a gap of dimension *b*, as shown in Fig. 1. The total width of the array is assumed to be *W* = *n* ⋅ (*a* + *b*), where *n* is the number of wires. Manipulating *a* and *b* and comparing the conductivity of the array with a 2D film of width *W* allow gaining insights into the impact of size quantization and, as will be shown, indicate a window of operation for which an array of 1D wires can outperform a 2D film despite the material loss associated with introducing “cuts” of width *b*.

To perform a quantitative analysis, we first consider graphene and then extend our calculation to a semiconductor with parabolic E(k) relation. Starting from the two-dimensional linear energy dispersion of graphene around the Dirac point, size quantization results in a set of one-dimensional modes as depicted in Fig. 1. The actual energy spacing between the individual 1D modes becomes larger (including the band gap) if *a* becomes smaller. At the same time, the number of modes M_1D_(E) becomes discrete in 1D. The band lineup under zero gate and drain bias conditions for each 1D wire is defined as the minimum of the lowest conduction band edge *E*_*C*0_ in the channel aligning with the source and drain Fermi levels in the contacts. All wires are assumed to respond in the same manner to the gate and drain field (see bottom part of Fig. 1). In case of the 2D graphene film, the threshold voltage is defined as the Dirac point. Through this approach of setting the threshold voltage to zero for both 1D wires and 2D films at the conduction band edge, a comparison of the device on-state needs to be concerned only with the gate voltage *V*_*GS*_ rather than the overdrive voltage *V*_*GS*_ − *V*_*th*_.

Under the conditions discussed above, the current through the device can be calculated using Landauer formalism. For ease of handling the analytical expressions, zero temperature conditions and ballistic transport in the quantum capacitance limit (QCL) are assumed. In the appendix, our calculations are extended toward 300 K (Additional file 1: Figure S1) showing that the analytical results obtained for *T* = 0 K as discussed in the following capture all relevant aspects and allow to understand the critical trends even quantitatively.

Within this model the electron current density (which is the only component considered) can be written as

1 $$ {I}_{2D}=W\frac{q}{h}{\displaystyle \underset{E_C}{\int }{M}_{2D}\left({f}_S-{f}_D\right)dE} $$

Here, *q* is the electron charge, *M*_2*D*_ is the number of propagating modes per unit width in the 2D device, ${M}_{2D}=\frac{h}{2}{D}_{2D}\cdotp {v}_{\mathrm{eff}}$, *D*_2*D*_ is the full density of states (including +k and −k-states), *v*_eff_ is the average electron velocity in transport direction, and *f*_*S*_ and *f*_*D*_ are the source and drain Fermi distributions, respectively.

If a positive gate bias is applied, the bottom of the conduction band is pulled down by exactly the amount of *qV*_*GS*_ because of the assumed operation in the quantum capacitance limit (QCL) [14, 15] and a positive drain voltage moves the drain Fermi level down by *qV*_*DS*_. Note that the assumption of operation in the QCL is justified for materials with low density of states when aggressively scaled gate oxides are considered. Furthermore, it should be noted that operation in the QCL is harder to achieve in the 2D case than for 1D due to the larger density of states in 2D. Thus, assuming that both 1D and 2D follow a one-to-one band movement with the gate voltage will potentially underestimate (but not overestimate) the amount of current by which the 1D current can surpass its 2D counterpart.

To calculate the current through the graphene transistor we note that (i) the current in a uniform 2D system is proportional to the device width W, (ii) the energy dispersion E(k) of graphene close to the Dirac point can be approximated by *E* = *v*_*f*_*ℏk*, and (iii) the density of states (DOS) is ${D}_{2D}=g\cdot 2\pi E/{h}^2{v}_f^2$, where *g* is the degeneracy factor, which is 4 for graphene—accounting for spin and valley degeneracy. To simplify the following calculations, we set *g* to 1. Furthermore, (iv) the average velocity in two dimensions is *v*_*eff*_ = 2*v*_*f*_/*π*. Under these assumptions, we find

2 $$ \left\{\begin{array}{cc}\hfill {I}_{2D}=\frac{W{q}^3}{h^2{v}_f}{V}_{GS}^2\hfill & \hfill {V}_{GS}<{V}_{DS}\hfill \\ {}\hfill {I}_{2D}=\frac{W{q}^3}{h^2{v}_f}\left(2{V}_{DS}{V}_{GS}-{V}_{DS}^2\right)\hfill & \hfill {V}_{GS}>{V}_{DS}\hfill \end{array}\right. $$

On the other hand, the current in a one-dimensional system is carried by 1D modes with discrete k-vector values in the quantization direction. For simplicity, we assume here hard wall potentials at the edges of the wires with width *a*, resulting in an energetic spacing between modes of *ΔE* = *hv*_*f*_/2*a*. This is a simple yet valid assumption if comparing our findings with results from first-principle calculation [16, 17]. Only modes in the energy interval between the source and the drain Fermi level contribute to the current. Moreover, *D*_1*D*_ ⋅ *v*_1*D*_ = 2/*h* for *g* = 1, independent of the actual energy dispersion. Assuming again zero temperature conditions and ballistic transport in the quantum capacitance limit as in the 2D case and noting that *v*_*eff*_ = *v*_*f*_ in the 1D case, the 1D current can be expressed as

3 $$ {I}_{1D}=n\frac{q}{h}{\displaystyle \int {M}_{1D}(E)\left({f}_S-{f}_D\right)}dE $$

Here, *n* = *W*/(*a* + *b*) is the number of wires, *m*(*E*) is the number of modes at each energy, *M*_1*D*_(*E*) = int[(*E* + *qV*_*GS*_)/*ΔE*] + 1 for *E* > − *qV*_*GS*_, and *m*(*E*) = 0 for *E* < − *qV*_*GS*_. Only currents due to electron flow in the conduction band are considered. It seems apparent that the current through an array of 1D structures cannot exceed the 2D current for finite *b*-values. Interestingly, this statement is *only* correct for *qV*_*GS*_ and *qV*_*DS*_ simultaneously being larger than *ΔE*. In fact, as will be discussed in the following for operation at sufficiently small bias conditions, Eq. 3 reveals higher current levels in 1D compared to the 2D transport case. For small bias conditions *qV*_*GS*_ < *ΔE*, *qV*_*DS*_ < *ΔE*, only one mode is conducting, and Eq. 3 simplifies to

4 $$ \left\{\begin{array}{cc}\hfill {I}_{1D}=\frac{W{q}^2}{\left(a+b\right)h}{V}_{GS}\hfill & \hfill {V}_{GS}<{V}_{DS}\hfill \\ {}\hfill {I}_{1D}=\frac{W{q}^2}{\left(a+b\right)h}{V}_{DS}\hfill & \hfill {V}_{GS}>{V}_{DS}\hfill \end{array}\right. $$

From Eq. 4, the conductance of each wire is independent of material choice *q*^2^/*h*. Comparing Eq. 4 with Eq. 2 now reveals a different trend. While the 2D current in Eq. 2 always shows a square-dependence on *V*_*GS*_ and *V*_*DS*_ at any biased condition, the 1D current in Eq. 4 exhibits a linear dependence on *V*_*GS*_ and *V*_*DS*_ at small bias values. A crossover between *I*_1*D*_(*V*_*GS*_, *V*_*DS*_) and *I*_2*D*_(*V*_*GS*_, *V*_*DS*_) is expected, with the 1D current being larger than the 2D current below this crossing point. It is worthwhile mentioning at this stage again that Eq. 4 holds true independent of material choice or the details of the E(k) relation as long as only one 1D mode is involved in current transport.

At this point, it is worthwhile reviewing the assumption of ballistic transport that has been made to allow obtaining the simple analytical expressions from above. For carbon nanotubes [8] and the cases of graphene and graphene nanoribbons [18–20], operation close to the ballistic limit has been reported, validating our approach. However, even in the case that scattering limits the current carrying capability of the device, Eq. 4 can still provide useful insights into the benefit of 1D transport. If the same scattering mechanisms prevail in the planar and ribbon device, the current in both cases is decreased by the same amount, and thus, the ratio between Eq. 2 and Eq. 4 remains unaltered, thus not impacting our analysis. Only if additional scattering in the ribbon case, e.g., due to the roughness of the edges, reduces the current in Eq. 4 more than in the 2D case, our analysis will be effected. In this case, the voltage range (see discussion below) over which 1D currents can be expected to exceed their 2D counterparts will be reduced by the same scaling factor that impacts the current in Eq. 4 due to scattering.

## Results and Discussion

### ON-State Performance

**Fig. 2:**
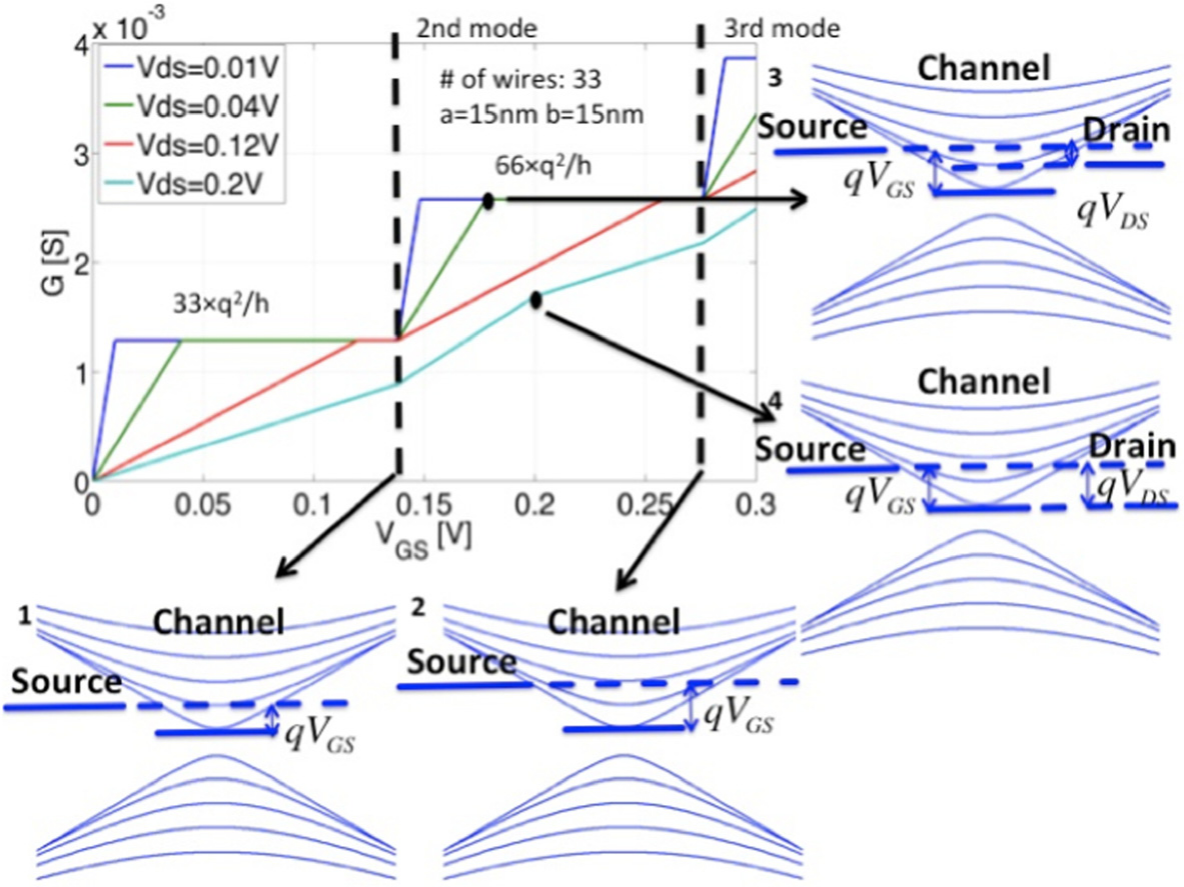
Calculated conductance versus gate voltage for different drain voltages. The diagrams indicate the relative position of the 1D subbands for the respective drain and gate voltage conditions. Diagrams 1 and 2 show the band alignment when the second, third mode starts to conduct. Diagram 3 shows the band alignment for current saturation. Diagram 4 shows the band alignment when only the first mode is saturated but not the second mode

Based on the above analytical framework, I–V characteristics have been calculated for various 1D transport scenarios. For all simulations, a width of *W* = 1 μm has been assumed. A set of conductance versus gate voltage curves for different drain voltages is plotted in Fig. 2. For the 1st subband, the conductance saturates at 33 ⋅ *q*^2^/*h*, where 33 is the total number of wires in the array, with the conductance contribution per wire for one subband being *q*^2^/*h* as expected from Eq. 4. The higher the drain voltage, the larger the gate voltage needed to reach the same conductance saturation level.

For *a* = 15nm, *ΔE* = *hv*_*f*_/2*a* ≅ 0.14 eV, which means that at *V*_*GS*_ = *m* ⋅ 0.14 V the (m + 1)th subband will start to conduct. This situation corresponds to band diagrams 1 and 2 that illustrate the second, third subband at gate voltages of 0.14 and 0.28 V aligned with the source Fermi level. For *V*_*DS*_ = 40 mV, *V*_*GS*_ = 0.18 V (diagram 3) maximum conductance through two subbands occurs since the minimum of the second subband is exactly by the amount of V_DS_ below the source Fermi level. Thus, even a further increase of gate voltage does not change the conductance until the third mode starts to conduct. Only when *V*_*GS*_ = *V*_*DS*_ + *m* ⋅ 0.14 V, the conductance through the (m + 1)th mode will saturate. Accordingly, for *V*_*DS*_ = 200 mV, *V*_*GS*_ = 200 mV (diagram 4), only the first mode in each wire has reached its saturation conductance of *q*^2^/*h* while the second mode has not. For gate voltage values below 200 mV, an increase in gate voltage leads to more conduction in both, the first and second mode. However, for gate voltages above 200 mV, an increase in gate voltage only leads to more conduction in the second mode. As a result, the slope of conductance versus gate voltage decreases at this point.

**Fig. 3:**
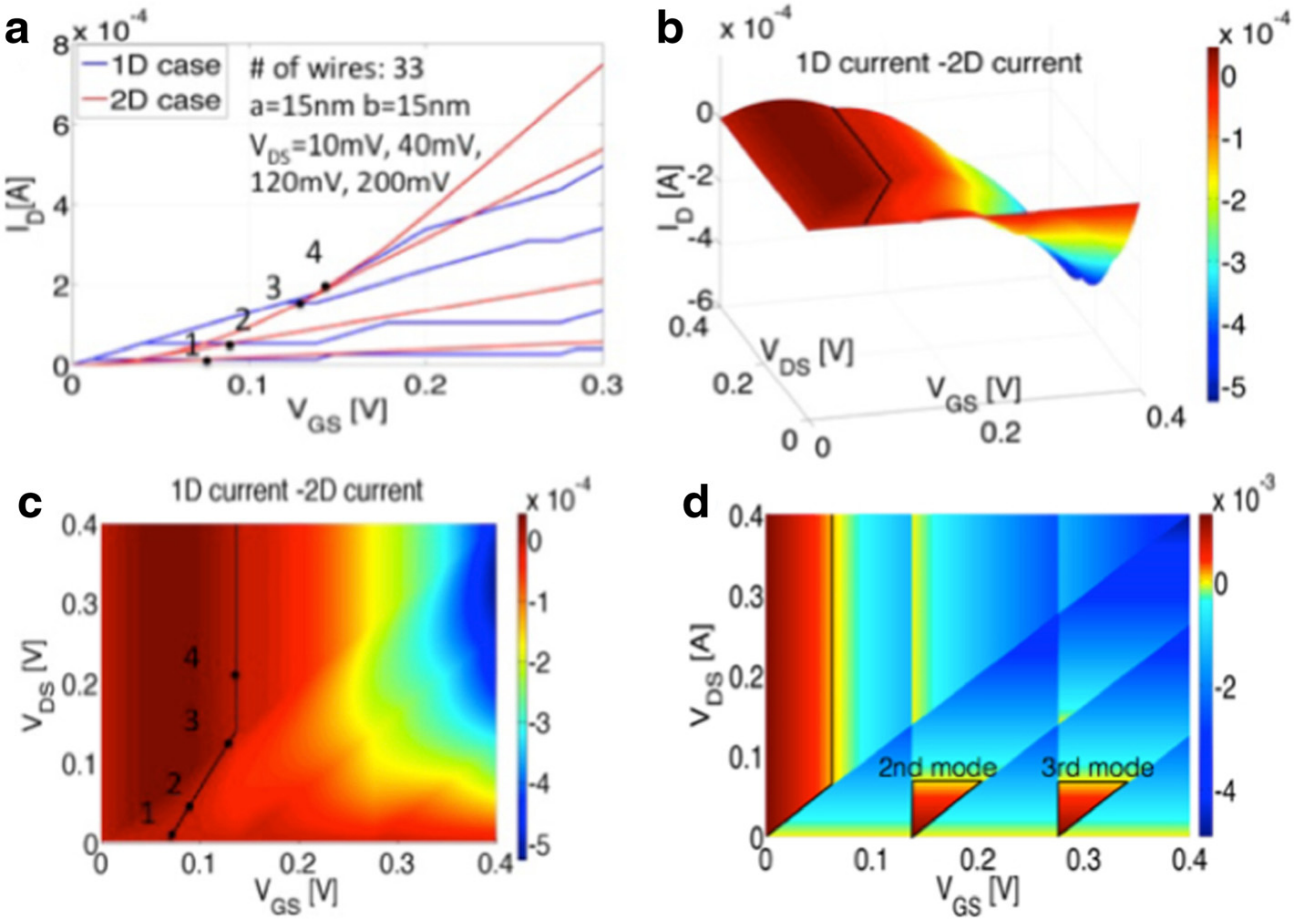
**a** I_D_ versus V_GS_ for both, the 1D and 2D case. **b** 3D plot of I_1D_–I_2D_ (*z*-axis: I_1D_–I_2D_, *x*-axis: V_GS_, *y*-axis: V_DS_), the *black line* indicates where the 1D current and the 2D current are equal. **c** 2D projection of **b. d** 3D plot for g_m1D_–g_m2D_ (*z*-axis: g_m1D_–g_m2D_, *x*-axis: V_GS_, *y*-axis: V_DS_), the *black line* indicates the region where 1D has a larger transconductance

Next, we compare the current levels in 1D and 2D. In Fig. 3a, both, the 1D current (blue) and the 2D current (red) are plotted as a function of gate voltage. It is clear that for small gate voltages, the 1D current can exceed the 2D counterpart as mentioned above in the context of Eq. 4. The crossing points are labeled from small drain voltages to large drain voltages as: 1, 2, 3, and 4. The corresponding positions are shown in Fig. 3c, and it is obvious that for drain voltages larger than *V*_*DS*_ = *ΔE*/*q* = 0.14 V, the position of the crossing point occurs at the same gate voltage of *V*_*GS*_ = *ΔE*/*q* = 0.14 V. For drain voltages below 0.14 V, the crossing points depend linearly on gate voltage, and *V*_*GS*_ approaches *ΔE*/2*q* = 0.07 V when the drain voltage tends to zero. Note that, as stated earlier, the assumption of operation in the QCL for both the 1D and 2D case is a conservative estimate that it will overestimate the band movement in the 2D case resulting in an underestimated gate voltage range for which 1D exhibits a larger current than 2D. In terms of transconductance *g*_*m*_, 1D can also exceed the 2D case for certain bias conditions (shown in Fig. 3d). Interestingly, this statement even holds true for large *V*_*GS*_ values as long as *V*_*DS*_ is small enough because of the onset of higher 1D modes.

Next, we will illustrate based on the DOS of 1D versus 2D how 1D currents can exceed their 2D counterparts. As discussed before, both 1D and 2D currents can be expressed as an energy integral of the number of conducting modes M_1D, 2D_(E) and the difference of the source and drain Fermi distributions. If we compare M_1D_ for a wire of width *a* with its counterpart in 2D: *a*M_2D_, one can derive the following expressions:

5 $$ {X}_{2D}=a{M}_{2D}=\frac{2Ea}{h{v}_f} $$

6 $$ {X}_{1D}={M}_{1D}=\operatorname{int}\left[\frac{2Ea}{h{v}_f}\right] $$

**Fig. 4:**
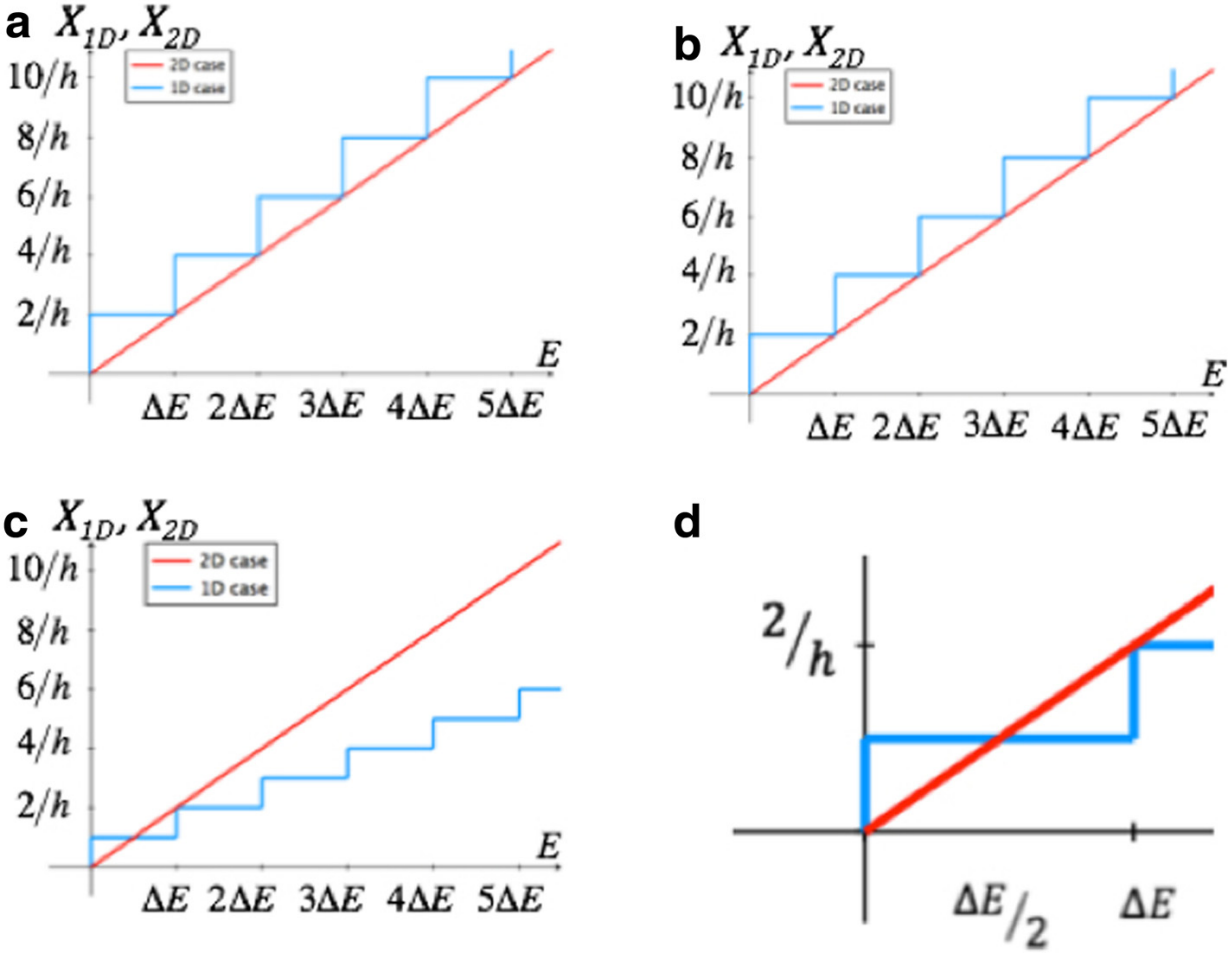
**a** *X*_*1D*_ and *X*_*2D*_ ,respectively, as defined in the text, **b** situation as in **a** after threshold voltage shift, **c** situation as in **a** after threshold voltage shift and accounting for material loss, and **d** zoom of **c**

Equation 5 was multiplied by the wire width *a* for a proper comparison of the 2D and 1D number of modes. Obviously, Eq. 6 is just the discrete version of Eq. 5 as shown in Fig. 4. Depending on the choice of threshold voltage (Δ*E* in case of Fig. 4a and zero in case of Fig. 4b), *X*_1*D*_ is smaller or larger than *X*_2*D*_. From Fig. 4b, one might conclude that the 1D case is always providing larger currents, but in reality, the material loss that is captured by the above-introduced parameter *b* needs to be considered as well. If we choose *a* = *b*, the material loss results in a scenario as depicted in Fig. 4c. Under these conditions, *X*_*1D*_ is larger than *X*_*2D*_ only for *V*_*GS*_ *< ΔE/q*. The exact conditions under which the 1D current can be larger than the 2D counterpart can be calculated by comparing ∫*X*_1*D*_*dE* and ∫*X*_2*D*_*dE*. As shown in Fig. 4c, d, *X*_1*D*_ is only larger for the energy region from 0 to *ΔE*/2, and for the integration range (0, *ΔE*), ∫*X*_1*D*_*dE* and ∫*X*_2*D*_*dE* are identical. This means that for *V*_*GS*_ larger than *ΔE*/*q* = 0.14*V*, the 2D current will be always larger which confirms the results in Fig. 3c. Equation 7 summarizes the conditions under which the 1D current exceeds the 2D one:

7 $$ \left\{\begin{array}{cc}\hfill q{V}_{GS}<\frac{\left(\varDelta E+q{V}_{DS}\right)}{2}\hfill & \hfill q{V}_{DS}\le \varDelta E\hfill \\ {}\hfill q{V}_{GS}<\varDelta E\hfill & \hfill q{V}_{DS}>\varDelta E\hfill \end{array}\right. $$

Note that Eq. 7 describes exactly the black line in Fig. 3c. If scattering is considered as discussed above, a scaling parameter that captures excess scattering in the ribbon case will have to be introduced in Eq. 7, which will reduce the voltage range over which the 1D currents are larger than the 2D ones.

**Fig. 5:**
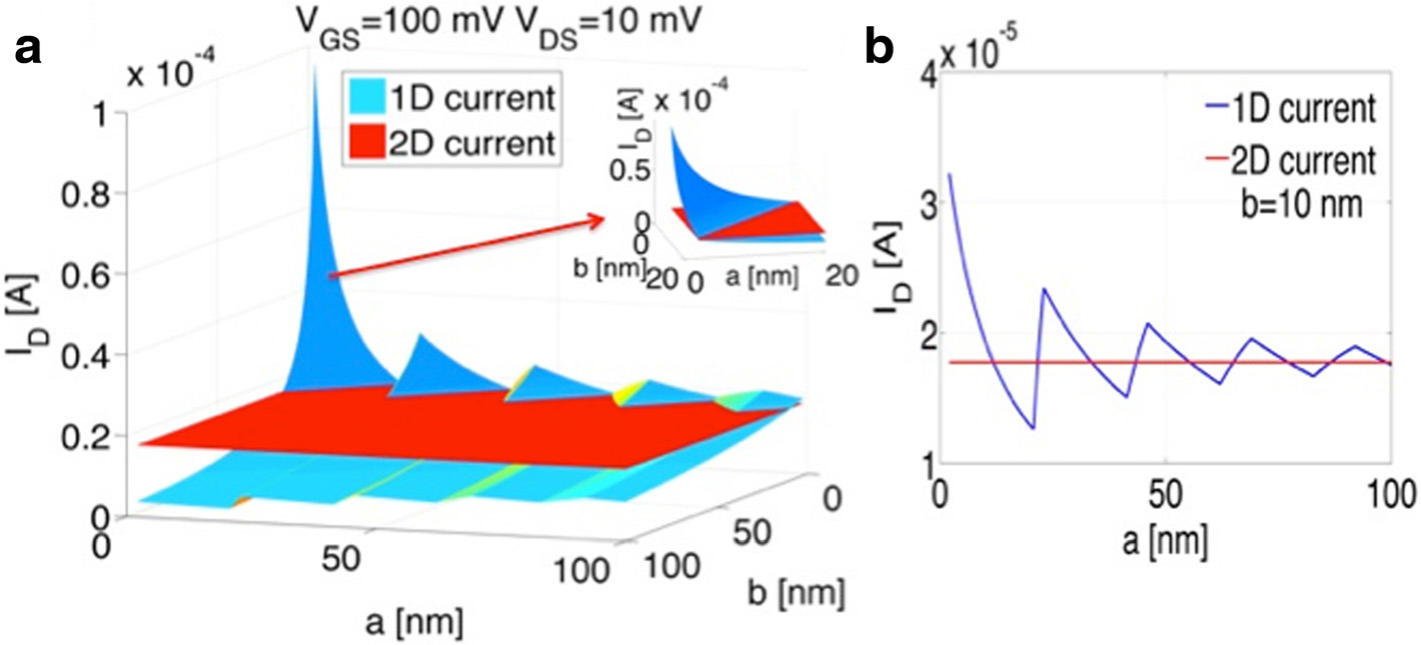
1D current and 2D currents are plotted as a function of **a** and **b** for a linear E(k)-relation

**Fig. 6:**
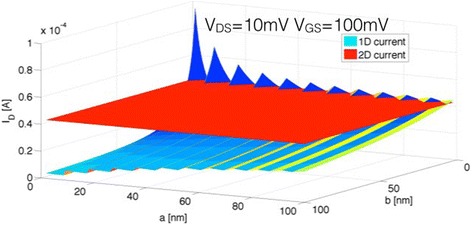
1D current and 2D currents are plotted as a function of **a** and **b** for a parabolic E(k)-relation

In the following, we want to focus on the interplay between *a* and *b*. As discussed above, the 1D current depends on both parameters, and depending on the introduced quantization conditions through *a* and the material loss through *b*, *I*_*1D*_ will exceed (or not) *I*_*2D*_. To illustrate this point, both the 1D and the 2D currents are plotted for different *a*, *b*-values in Fig. 5 for a linear and in Fig. 6 for a parabolic energy dispersion.

Since in general, different E(k) dispersion relations impact the above analysis only in so far that the density of states and energy quantization *ΔE* is changed, both, Figs. 5 and 6 show qualitatively the same dependences. While the energy dispersion impacts the values of the parameters *a*, *b*, *V*_*DS*_, and *V*_*GS*_ for which the 1D current can exceed the 2D counterpart, the general trends described above prevail. In particular, Eqs. 1, 3, and 4 are valid independent of the exact material choice. For the details of how Eq. 2 and the number of 1D modes *m*(*E*) are modified under the assumption of a parabolic energy dispersion, see the appendix.

For (*a*,*b*) = (0,0), *I*_*1D*_ becomes infinite since the number of wires *W*/(*a + b*) contributing *q*^*2*^/*h* to the conductance becomes infinite. Also, as expected, small *b*-values are in general desirable to reduce the amount of material loss. The *a*-dependence is somewhat more surprising. In fact, we find a non-monotonic dependence of the 1D current with *a* for constant *b* as shown in Fig. 5b. Two effects need to be considered when *a* increases. On one hand, the number of contributing wires decreases with increasing *a* for fixed *W* and *b*. This results in a *I*_*1D*_*∝ 1/a* trend as depicted in Fig. 5b. On the other hand, increasing *a* changes the quantization conditions per wire and decreases the mode spacing *ΔE*. The sharp increases in current around 21, 42, and 63 nm are a result of this effect. For these *a*-values, Δ*E* is 100, 50, and 25 meV, respectively. From the discussion above, the number of contributing modes at source Fermi level is simply int(*qV*_*GS*_/*ΔE* + 1) which implies that for *a* = 21, 42, and 63 nm, the second, third, and fourth mode starts conducting for a *V*_*GS*_ of 100 mV. The amount of current change at the onset of the *n*th mode is proportional to *n*/*(n−*1*)* which implies a current increase by a factor of 2, 1.5, and 1.33 at *a* = 21, 42, and 63 nm, respectively, consistent with Fig. 5b.

### Off-State Performance

**Fig. 7:**
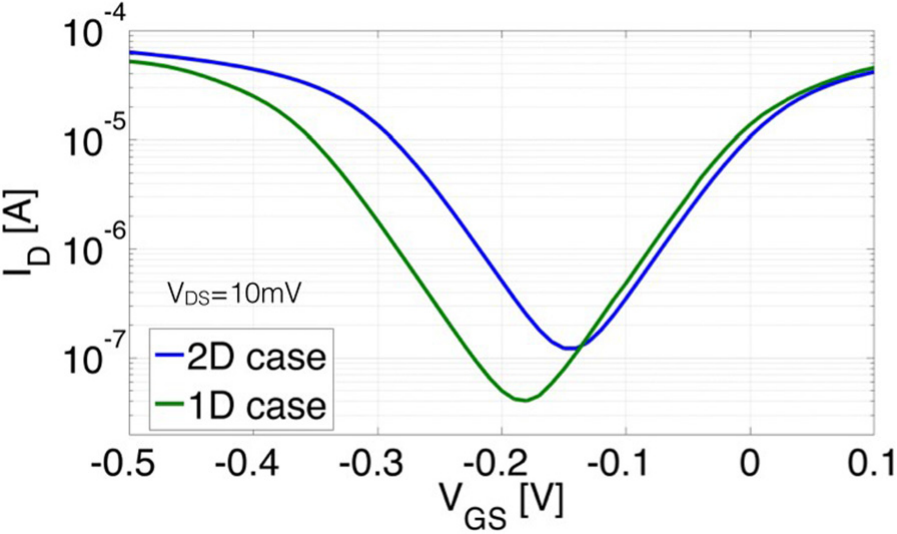
Transfer characteristics for the 1D and 2D case for a parabolic E(k)-relation. As above, a width of *W* = 1 μm and *a* = 10 nm, *b* = 4 nm has been assumed

So far, the discussion had only been concerned with the on-state performance of an array of 1D wires in comparison with their 2D counterpart. In this section, we will discuss that the abovementioned benefits of a higher on-current in 1D for certain parameters do in fact *not* come at the expense of a deteriorated off-state performance of the device. In order to come to this conclusion, currents through both, the conduction and valence band need to be considered. If a band gap is assumed in a semiconductor with parabolic bands (see also Fig. 6), size quantization increases the energetic spacing between the maximum of the valence band and the minimum of the conduction band for the 1D case. To quantify the impact of this band gap change, the above condition about zero Kelvin operation needs to be revised since, otherwise, an infinitely steep inverse subthreshold slope and, accordingly, an infinite on/off-current ratio would make the comparison between the 2D and 1D scenario meaningless. Figure 7 shows transfer characteristics for both, the 1D and the 2D case at 300 K. As apparent from the plot, the quantization conditions in the nanowires result in a larger band gap that leads to a larger on/off-current ratio, i.e., in particular, a substantially lower minimum current level as shown in Fig. 7.

## Conclusions

In conclusion, we have presented in this article a simple analysis focusing on both the on-current in arrays of one-dimensional wires if compared to a two-dimensional structure of similar dimensions. Different from general expectations, an array of 1D structures can outperform the current in a 2D system if threshold voltages are properly adjusted, even under room temperature operation. The above discussion provides a simple guide to perform similar comparisons for other material systems and device structures.

## Additional file

Additional file 1:
**On the Current Drive Capability of Low Dimensional Semiconductors -1D versus 2D –. ** The current calculation for parabolic E-k relation and the disscusion of temperture dependence is shown in the addtional file. **Figure S1.** shows the difference of 1D and 2D currrent under T=0k & T=300K.

## References

[1] Hisamoto D, Lee WC, Kedzierski J, Takeuchi H, Asano K, Kuo C, Anderson E, King TJ, Bokor J, Hu C (2000). FinFET—a self-aligned double-gate MOSFET scalable to 20 nm. IEEE Trans Electron Devices.

[2] Huang XJ, Lee WC, Kuo C, Hisamoto D, Chang LL, Kedzierski J, Anderson E, Takeuchi H, Choi YK, Asano K, Subramanian V, King TJ, Bokor J, Hu C (2001). Sub-50 nm p-channel FinFET. IEEE Trans Electron Devices.

[3] Kedzierski J, Ieong M, Nowak E, Kanarsky TS, Zhang Y, Roy R, Boyd D, Fried D, Wong HSP (2003). Extension and source/drain design for high-performance FinFET devices. IEEE Trans Electron Devices.

[4] Kedzierski J, Nowak E, Kanarsky T, Zhang Y, Boyd D, Carruthers R, Cabral C, Amos R, Lavoie C, Roy R, Newbury J, Sullivan E, Benedict J, Saunders P, Wong K, Canaperi D, Krishnan M, Lee KL, Rainey BA, Fried D, Cottrell P, Wong HSP, Ieong M, Haensch W. Metal-gate FinFET and fully-depleted SOI devices using total gate silicidation. IEDM. 2002;247–250. doi:10.1109/IEDM.2002.1175824.

[5] Lansbergen GP, Rahman R, Wellard CJ, Woo I, Caro J, Collaert N, Biesemans KG, Hollenberg LCL, Rogge S (2008). Gate-induced quantum-confinement transition of a single dopant atom in a silicon FinFET. Nature Phys.

[6] Pei G, Kedzierski J, Oldiges P, Ieong M, Kan ECC (2002). FinFET design considerations based on 3-D simulation and analytical modeling. IEEE Trans Electron Devices.

[7] Giannetta RW, Olheiser TA, Hannan M, Adesida I, Melloch MR (2005). Conductance quantization and zero bias peak in a gated quantum wire. Phys E.

[8] Javey A, Guo J, Wang Q, Lundstrom M, Dai HJ (2003). Ballistic carbon nanotube field-effect transistors. Nature.

[9] Franklin AD, Chen Z (2010). Length scaling of carbon nanotube transistors. Nature Nanotech.

[10] Frank DJ, Dennard RH, Nowak E, Solomon PM, Taur Y, Wong HSP (2001). Device scaling limits of Si MOSFETs and their application dependencies. Proc IEEE.

[11] Guo J, Datta S, Lundstrom M (2004). A numerical study of scaling issues for Schottky-barrier carbon nanotube transistors. IEEE Trans Electron Devices.

[12] Shin M, Lee J, Ahn C (2008). Simulation study of the scaling behavior of top-gated carbon nanotube field effect transistors. J Nanosci Nanotech.

[13] Meric I, Dean CR, Young AF, Baklitskaya N, Tremblay NJ, Nuckolls C, Kim P, Shepard KL (2001). Channel length scaling in graphene field-effect transistors studied with pulsed current–voltage measurements. Nano Lett.

[14] Chen ZH, Appenzeller J. Mobility extraction and quantum capacitance impact in high performance graphene field-effect transistor devices. IEDM. 2008;509–512. doi:10.1109/IEDM.2008.4796737

[15] Luryi S (1988). Quantum capacitance devices. Appl Phys Lett.

[16] Gunlycke D, White CT (2008). Tight-binding energy dispersion of armchair-edge graphene nanostrips. Phys Rev B.

[17] Brey L, Fertig HA (2006). Electronic states of graphene nanoribbons studied with Dirac equation. Physical Rev B.

[18] Baringhaus J, Ruan M, Elder F, Tejeda A, Sicot M, Ibrahimi AT, Li AP, Jiang Z, Conrad EH, Berger C, Tegenkamp C, Heer DWA (2014). Exceptional ballistic transport in epitaxial graphene nanoribbons. Nat.

[19] Fang T, Konar A, Xing HL, Jena D (2008). Mobility in semiconducting graphene nanoribbons: phonon, impurity, and edge roughness scattering. Physical Rev B.

[20] Shin YS, Son YJ, Jo MH, Shin YH, Jang HM (2011). High-mobility graphene nanoribbons prepared using polystyrene dip-pen nanolithography. J Am Chem Soc.

