# Supplementary material for: On the Current Drive Capability of Low Dimensional Semiconductors: 1D versus 2D
Source: Nanoscale Res Lett. 2015 Oct 29;10:425. doi: 10.1186/s11671-015-1134-6 (PMC4626470; doi:10.1186/s11671-015-1134-6)
Supplement: Additional file 1: — On the Current Drive Capability of Low Dimensional Semiconductors -1D versus 2D –. The current calculation for parabolic E-k relation and the disscusion of temperture dependence is shown in the addtional file. Figure S1. shows the difference of 1D and 2D currrent under T=0k & T=300K. [file 11671_2015_1134_MOESM1_ESM.docx]

**Supplementary Information for: On the Current Drive Capability of Low Dimensional Semiconductors**

- **1D versus 2D –**

Y. Zhu* and J. Appenzeller

Birck Nanotechnology Center, Purdue University, West Lafayette, Indiana 47907, United States.

**Calculation for parabolic E-k relation**

For a parabolic E(k) relation: $E=p^{2}/2m^{*}$, where $m^{*}$is the effective electron mass in channel. In 2D, the density of states and effective velocity can be described by:

$D_{2D}=g\times\frac{2\pi m^{*}}{h^{2}}$ (S1)

$v_{eff}=\frac{2}{\pi}\sqrt{\frac{2E}{m^{*}}}$ (S2)

If we assume *g* to be 1 and only consider the electron currents, *I_2D_* becomes:

$\left\{\begin{matrix} I_{2D}=\frac{4\sqrt{2m^{*}}Wq^{\frac{5}{2}}}{3h^{2}}V_{GS}^{\frac{3}{2}} & V_{GS}<V_{DS} \\ I_{2D}=\frac{4\sqrt{2m^{*}}Wq^{\frac{5}{2}}}{3h^{2}}[V_{GS}^{\frac{3}{2}}-(V_{GS}-V_{DS})^{\frac{3}{2}}] & V_{GS}>V_{DS} \end{matrix}\right.$ (S3)

Which replaces the set of equations (2) from above for the parabolic E(k)-case. In 1D on the other hand as discussed abovefor *g*=1: $D_{1D}v_{1D}=2/h$. Using the same size quantization conditions: $\Delta k=\pi/a$. The current in the 1D case becomes:

$I_{1D}=\frac{qW}{h(a+b)}(\int_{-qV_{GS}}^{0} m(E)dE-\int_{-qV_{GS}}^{-qV_{DS}} m(E)dE)$ (S4)

With the number of modes$m(E)=\mathrm{int}(\sqrt{8m^{*}a^{2}(E+qV_{GS})/h^{2}}+1)$.

**Temperature Dependence**

The impact of finite temperature is included by the $kt$-term within the Fermi distribution. In this case the current equation becomes:
 $I_{1D}=\frac{Wq}{(a+b)h}\int_{Ec}^{\infty} M_{1D}(E)(f_{S}-f_{D})dE$ (S5)

$I_{2D}=W\frac{q}{h}\int_{Ec}^{\infty} M_{2D}(E)(f_{S}-f_{D})dE$ (S6)

From the result shown in fig. S1 it is clear that the temperature does not impact our quantitative conclusion about the 1D current surpassing its 2D counterpart substantially.


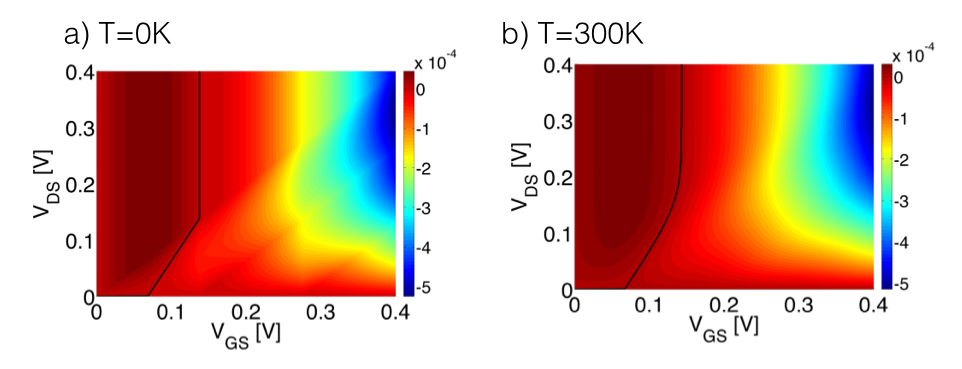


Figure S1: a) Plot of I_1D_-I_2D_ for different V_GS_ and V_DS_ biases at T=0K b) Plot of I_1D_-I_2D_ for different V_GS_ and V_DS_ biases at T=300K.
